# Assessing microvascular invasion in HBV-related hepatocellular carcinoma: an online interactive nomogram integrating inflammatory markers, radiomics, and convolutional neural networks

**DOI:** 10.3389/fonc.2024.1401095

**Published:** 2024-09-16

**Authors:** Yun Zhong, Lingfeng Chen, Fadian Ding, Wenshi Ou, Xiang Zhang, Shangeng Weng

**Affiliations:** ^1^ Department of Hepatobiliary and Pancreatic Surgery, The First Affiliated Hospital, Fujian Medical University, Fuzhou, China; ^2^ Fujian Abdominal Surgery Research Institute, The First Affiliated Hospital, Fujian Medical University, Fuzhou, China; ^3^ Department of Hepatobiliary and Pancreatic Surgery, National Regional Medical Center, Binhai Campus of the First Affiliated Hospital, Fujian Medical University, Fuzhou, China; ^4^ Fujian Provincial Key Laboratory of Precision Medicine for Cancer, The First Affiliated Hospital, Fujian Medical University, Fuzhou, China

**Keywords:** hepatocellular carcinoma, microvascular invasion, radiomics, convolutional neural network, inflammation marker

## Abstract

**Objective:**

The early recurrence of hepatocellular carcinoma (HCC) correlates with decreased overall survival. Microvascular invasion (MVI) stands out as a prominent hazard influencing post-resection survival status and metastasis in patients with HBV-related HCC. The study focused on developing a web-based nomogram for preoperative prediction of MVI in HBV-HCC.

**Materials and methods:**

173 HBV-HCC patients from 2017 to 2022 with complete preoperative clinical data and Gadopentetate dimeglumine-enhanced magnetic resonance images were randomly divided into two groups for the purpose of model training and validation, using a ratio of 7:3. MRI signatures were extracted by pyradiomics and the deep neural network, 3D ResNet. Clinical factors, blood-cell-inflammation markers, and MRI signatures selected by LASSO were incorporated into the predictive nomogram. The evaluation of the predictive accuracy involved assessing the area under the receiver operating characteristic (ROC) curve (AUC), the concordance index (C-index), along with analyses of calibration and decision curves.

**Results:**

Inflammation marker, neutrophil-to-lymphocyte ratio (NLR), was positively correlated with independent MRI radiomics risk factors for MVI. The performance of prediction model combined serum AFP, AST, NLR, 15 radiomics features and 7 deep features was better than clinical and radiomics models. The combined model achieved C-index values of 0.926 and 0.917, with AUCs of 0.911 and 0.907, respectively.

**Conclusion:**

NLR showed a positive correlation with MRI radiomics and deep learning features. The nomogram, incorporating NLR and MRI features, accurately predicted individualized MVI risk preoperatively.

## 1 Introduction

Liver cancer, the fifth most common malignant tumor, ranks fourth in mortality of cancer (1). Hepatocellular carcinoma (HCC) accounts for approximately 90% of the cases of liver cancer worldwide (2). At least 50% cases of HCC worldwide were caused by hepatitis B virus (HBV). In China, chronic HBV infection is also the main cause of HCC (3, 4).

At present, the main treatment options for HCC encompass liver resection, liver transplant, and transcatheter arterial chemoembolization (TACE) (5). Post-surgical resection, the annual recurrence rate of hepatocellular carcinoma (HCC) is at least 10%, escalating to 70–80% within a five-year period. The recurrent HCC tumors may probably progress into incurable, advanced-stage disease in most patients (6). So accurately identifying high-risk patients, estimating the prognosis of those with HCC, and extending survival time are of critical importance in clinical practice.

Microvascular invasion (MVI) serves as a significant independent prognostic factor for patients with hepatocellular carcinoma (HCC) following curative treatments, including surgical resection, liver transplantation, or alternative therapeutic interventions (7). Under microscopic examination, microvascular invasion (MVI) is characterized by the presence of neoplastic cell clusters within the lumina of endothelial-lined vascular channels, including those of the portal and hepatic venous systems (8). Recent evidence suggested that MVI might be the first step in the occurrence of intra-hepatic or systemic metastasis of HCC (9). Several retrospective studies suggest an association between inflammation and MVI in HBV-HCC patients (10). Inflammatory environment may increase hepatic microvascular permeability so that cancer cells can invade through the blood vessel wall (11). The occurrence of microvascular invasion (MVI) signifies the infiltration of cancer cells into the vasculature, heralding the potential onset of metastasis.

There has been an increasing interest in predicting MVI through preoperative data. Recent studies have highlighted the potential utility of blood-cell-inflammatory markers as non-invasive predictors of MVI (12, 13). These markers, including neutrophil-to-lymphocyte ratio (NLR), platelet-to-lymphocyte ratio (PLR), and systemic immune-inflammation index (SII), reflect the patient’s systemic inflammatory response, which is closely associated with tumor progression and metastasis. It has been previously observed that an elevated NLR is correlated with an increased incidence of MVI in HCC patients (14). Furthermore, the SII, which combines neutrophil, lymphocyte, and platelet counts, has shown superior predictive accuracy for MVI compared to individual markers (15). The integration of these inflammatory markers into clinical practice could enhance the preoperative assessment of MVI, allowing for more tailored and effective treatment plans for HCC patients.

As known, preoperative images are also important preoperative data. Radiomics, defined as the transformation of digital medical images into high-dimensional, analyzable data through advanced computational techniques, facilitates the exhaustive extraction and quantification of data from standard radiological imagery, thereby yielding critical insights into the cancer phenotype and the tumor microenvironment (16). Recent studies suggest an association between some radiographic features and local inflammatory status and vascular response in tumor (17). Li Yang reported that a nomogram incorporating radiomic features extracted from hepatobiliary phase (HBP) imaging demonstrated efficacy in the preoperative prognostication of MVI risk in HCC patients (18). PENG LIU has substantiated that radiomic analysis of computed tomography images exhibits a definitive predictive value for MVI in solitary HCC with a dimension less than or equal to 5 cm (19). Gadobenate dimeglumine-enhanced MRI imaging carries additional information on tumors than computed tomography, which can also reflect changes in the tumor micro-environment (20).

Deep learning with convolutional neural networks (CNNs) was applied to extract the inherent features of input data automatically (21). Recently, Residual Neural Network, a classical deep learning model, has been widely used for 3D imaging data analysis in medical field including MRI (22). Li et al. utilized a six-layer CNN to extract features from MR images to classify low grade gliomas and found an improvement on the traditional radiomics (23). Unlike traditional computed features, deep features retain a large amount of the global spatial information. So far, very little attention has been paid to the role of blood-cell-inflammatory markers combined with radiomics and deep learning features from preoperative MRI in predicting MVI.

In this study, we aimed to integrate preoperative MRI characteristics with inflammatory markers to create and confirm a new predictive nomogram for the preoperative estimation of MVI in HBV- related HCC. This nomogram facilitates the preoperative determination of the individualized risk of MVI in HCC patients, which is especially beneficial for categorizing patients into appropriate treatment groups.

## 2 Materials and methods

### 2.1 Patients and follow-up

**Figure 1:**
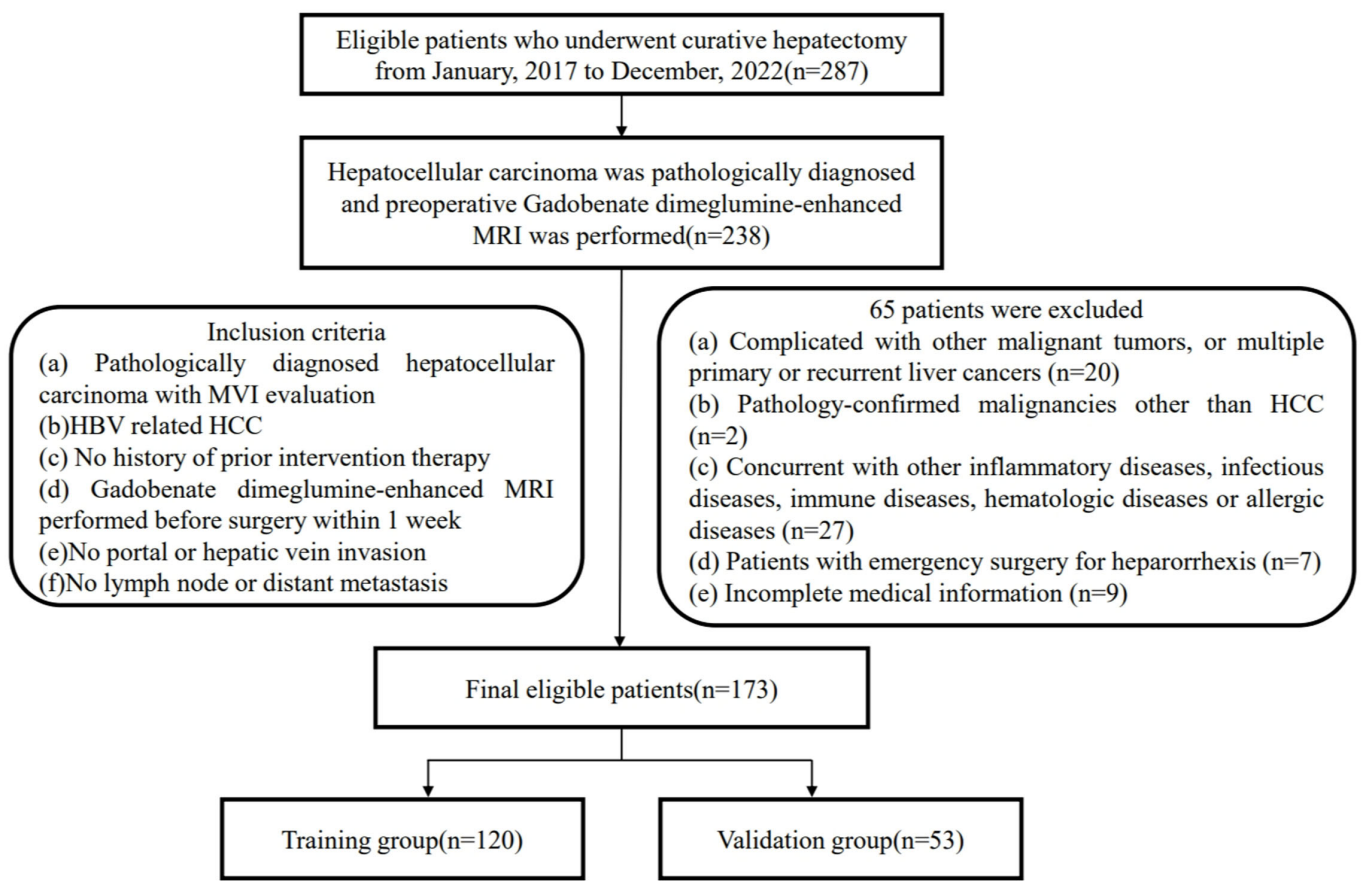
Flow chart of the patient enrollment process.

Ethical approval was obtained for this retrospective study, and the requirement for informed consent was waived. Patients were retrospectively collected from January 1, 2017 to November 31, 2022 (The First Affiliated Hospital of Fujian Medical University). [MTCA, ECFAH of FMU[2015] No.084-1] The inclusion criteria were: (a) Pathologically diagnosed hepatocellular carcinoma with MVI evaluation; (b) HBV related HCC; (c) Without history of prior intervention therapy; (d) Gadobenate dimeglumine-enhanced MRI was performed before surgery within 1 week; (e) No portal or hepatic vein invasion; (f) No lymph node or distant metastasis. The exclusion criteria were: (a) Complicated with other malignant tumors, and multiple primary or recurrent liver cancer; (b) Pathology-confirmed malignancies were not HCC; (c) Combined with other infectious diseases, immune diseases, hematologic diseases or allergic diseases; (d) Patients with emergency surgery for heparorrhexis; (e) Incomplete medical information. The ultimate composition of the patient cohort encompassed a total of 173 individuals (150 men and 23 women). The training group included 120 patients, and 52 patients were allocated to the validation group. The process for patient inclusion was detailed in the flowchart provided in **Figure 1**. Recurrence-free survival (RFS) time referred to the time interval from surgery to the date of recurrence, death or the last follow-up. The RFS was analyzed using the Kaplan-Meier method to estimate the survival distribution in this population. The Log-rank test was employed to compare the survival differences between MVI positive and MVI negative groups.

### 2.2 Laboratory and pathology data acquisition

Clinical-pathological baseline data were systematically extracted from our institution’s medical archives. Clinical features included age, gender, BMI, neutrophil, lymphocytes, hemoglobin, platelets, serum albumin, alkaline phosphatase (ALP), aspartate aminotransferase (AST), alanine aminotransferase (ALT), γ-glutamyl transpeptidase (γ-GT), AFP, the Systemic Immune-inflammation Index (SII), the Neutro-phil-to-Lymphocyte Ratio (NLR), the Platelet-to-Lymphocyte Ratio (PLR). MR image characteristics including enhancement pattern, peritumoral enhancement on artery phase, radiologic capsule on delay phase, peritumoral hypointensity were analyzed by 2 experienced radiologists. The pathological attributes, including tumor count, MVI presence, tumor size, Edmondson-Steiner grading, and cirrhosis status in non-tumorous liver tissue, were assessed by 2 experienced pathologists. Laboratory analyses consisted of common hematology tests were performed within one week prior to the surgical intervention.

### 2.3 Magnetic resonance imaging data acquisition

**Figure 2:**
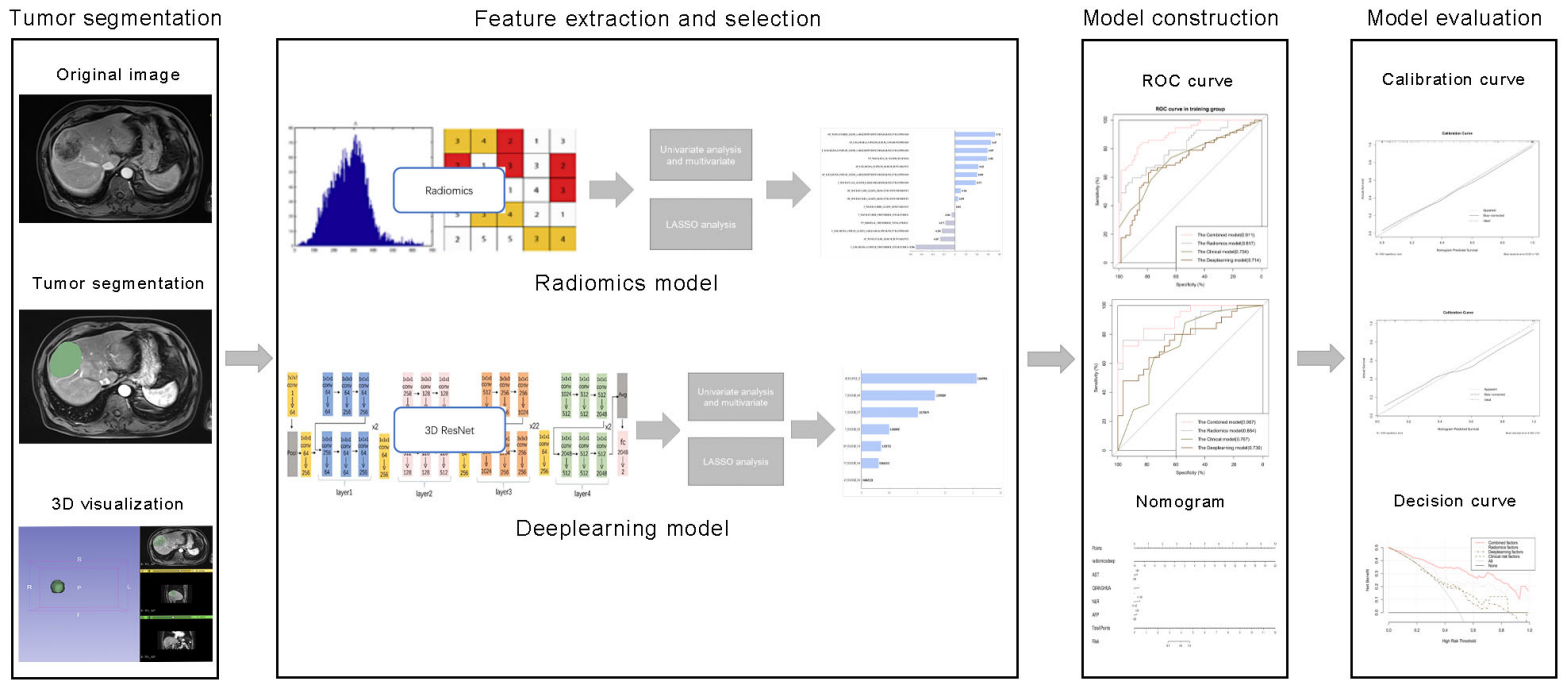
Comprehensive workflow diagram of the prediction model. Tumor segmentation in MR images is the first step. After that, MRI feature extraction was conducted separately via radiomics and neural convolutional networks. Student's t-test, Mann-Whitney U test and least absolute shrinkage and selection operator (LASSO) were used to feature selection, sequentially. The receiver operating characteristic (ROC) curve and the area under the curve (AUC) were calculated to evaluate the prediction efficiency of the radiomic features. Finally, a nomogram was developed and evaluated.

all study patients underwent gadobenate dimeglumine-enhanced MR imaging using 3.0-Tesla MR scanners (Magnetom Skyra Siemens Healthcare). Conventional MRI parameters were as follows: repetition time (TR)/echo time (TE), 6000/125 ms; number of excitation (NEX), 1; field of view (FOV), 20 mm; slices, 22; slice thickness, 3 mm. Gadopentetate dimeglumine was administrated with a dose of 0.1 mmol/kg, followed by a 20-mL continuous saline flush. Imaging sequences included T2-weighted imaging with fat suppression, T1-weighted imaging, and contrast-enhanced T1-weighted im-aging obtained at 20–30 s (by monitoring, the scan is triggered when the contrast agent reaches the ascending aorta), 70–90 s, 100–120 s, and 160–180 s respectively after contrast medium injection. That included a transverse arterial phase, transverse portal venous phase and transverse delayed phase. Image characteristics including enhancement pattern, peritumoral enhancement on artery phase, radiologic capsule on delay phase and peritumoral hypointensity were analyzed by 2 experienced radiologists (**Figure 2**).

### 2.4 Workflow of radiomics analysis

The radiomics analysis workflow encompassed the segmentation of the tumor, the extraction and selection of features, followed by the construction and evaluation of the predictive model (**Figure 2**). 1223 candidate texture parameters were extracted, including shape_LeastAxisLength, shape_MeshVolume,shape_MinorAxisLength, glrlm_RunEntropy, ngtdm_Coarseness, gldm_DependenceNonUniformity and so on. All feature extraction was implemented by Python (Version 3.10.5).

#### 2.4.1 Tumor segmentation

**Figure 3:**
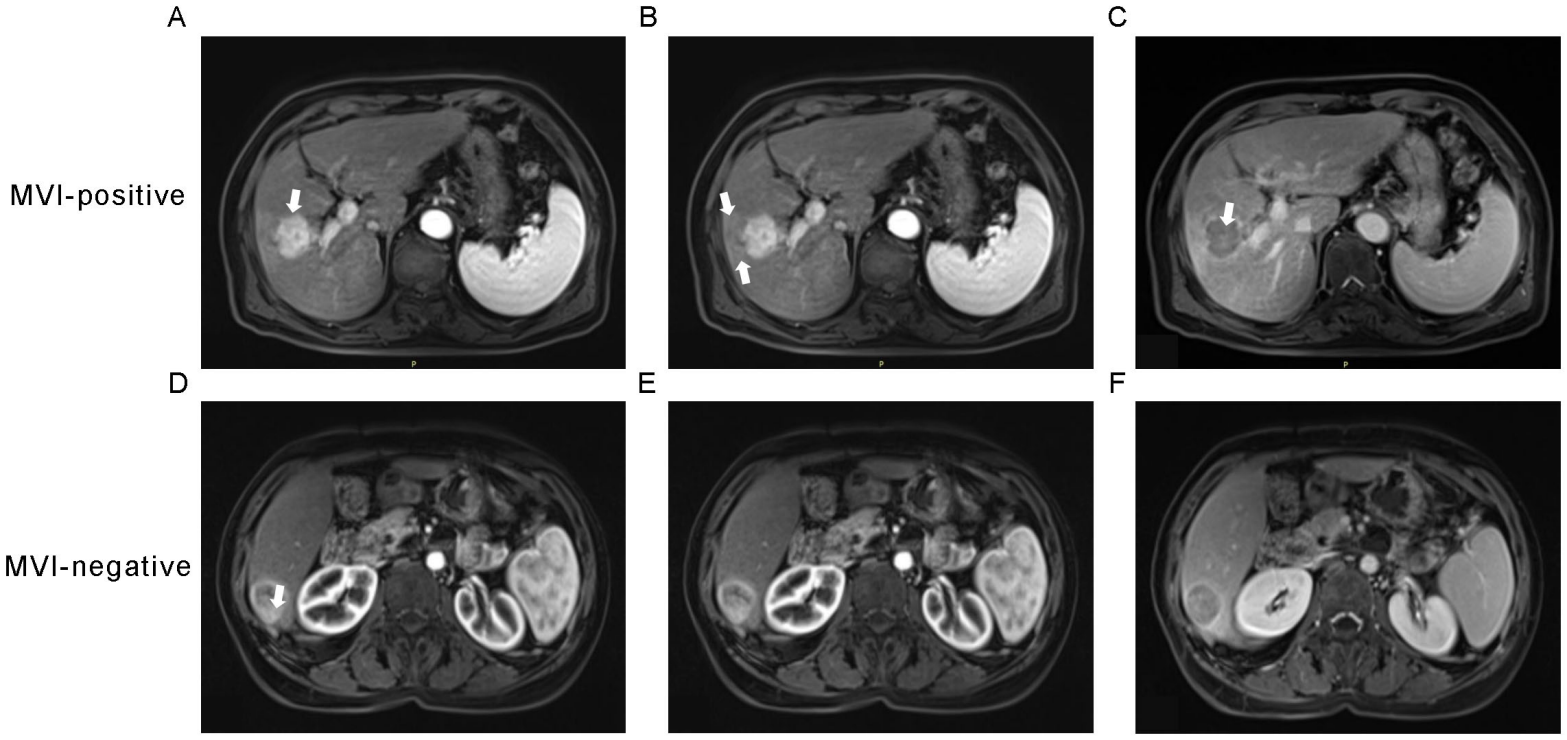
Characteristic MRI images of microvascular invasion (MVI)-positive and MVI-negative hepatocellular carcinoma. **(A, B)** The axial arterial phase image shows a non-smooth hype vascular tumor (arrow) with peritumoral enhancement (arrows). **(C)** The delayed phase image exhibits a rapid low signal intensity (arrow) within the tumor. **(D, E)** The arterial phase image demonstrates a smooth tumor, accompanied by **(F)** a radiologic capsule in the delayed phase.

The MRI images of the patients were exported in DICOM format. Blind to the pathological findings, the radiologist, boasting three years of expertise in abdominal imaging, employed the 3D-Slice software (www.slice.org) to demarcate the region of interest (ROI) on each slice encompassing the tumor. Regions of interest (ROIs) were drawn on all arterial phase (AP), venous phase (VP), delayed phase (DP) and T2-weighted (T2) images slice-by-slice for each patient (**Figure 3**).

#### 2.4.2 Feature extraction

Features, including radiomics features and deep learning features, were extracted through Python package Pyradiomics and trained 3D ResNet, respectively. 1223 radiomics texture parameters were extracted, including first-order features and texture features in every phase. In another way, 3D ResNet, a pre-trained deep learning architecture, was utilized to harvest 512 deep features from the ROIs of the images for each patient in every MRI sequence. All feature extraction was implemented by Python (Version 3.10.5).

#### 2.4.3 Model construction and evaluation

The clinical model was constructed using features that demonstrated a statistical significance with p-values less than 0.05 in the multivariate logistic regression analysis. We built a radiomics model and a deep learning model using features that were extracted by the above method. A combined model was constructed by integrating elements from the clinical model with the most effective signatures from radiomics and deep learning models. The efficiency of the radiomic feature-based predictions was assessed by computing the receiver operating characteristic (ROC) curve and the corresponding area under the curve (AUC). The area under the curve (AUC) was ascertained along with its 95% confidence interval (CI), in addition to quantifying the sensitivity, specificity, and precision of the model. A nomogram was constructed to visually represent the combined model. The discriminative efficacy of the nomogram was quantified employing Harrell’s concordance index (C-index). Calibration curves were generated and analyzed to assess the diagnostic concordance of the nomogram within the training and validation cohorts. Decision curve analysis was employed to ascertain the clinical utility of the nomogram. A web-based calculator for the dynamic prediction of MVI was created utilizing the “shiny” and “DynNom” packages (https://www.shinyapps.io/).

### 2.5 Evaluation of NLR and radiomics and deep features

The correlation between the NLR and extracted features were represented by “ggpubr” and “ggExtra” R packages. Besides, we explored the relationship between the NLR and risk score of every model by Pearson correlation analysis.

### 2.6 Statistical analysis

All statistical analyses were conducted utilizing IBM SPSS Statistics (Version 24.0), Python (Version 3.10.5), and R (Version 4.3.1) software packages. A two-tailed p value less than 0.05 was considered statistically significant.

## 3 Results

### 3.1 Baseline characteristics and survival curve

The baseline characteristics of the study participants were presented in **Supplementary Table S1**. These data provided a comprehensive overview of the demographic, clinical, and biochemical characteristics at the onset of the study. There was no significant difference in the incidence of MVI between the training and validation groups. Meanwhile, we conducted a follow-up study involving 111 patients from this population and plotted the survival curve (**Supplementary Figure S1**). This survival curve demonstrated that MVI was a major risk factor for HCC recurrence.

### 3.2 Clinical feature analysis

The clinical and MR image characteristics collected from MVI-negative and MVI-positive groups were presented in **Table 1**. Youden index was used to find the best cut-off value of inflammation markers (**Table 2**). NLR, AST, serum AFP levels, and peritumoral enhancement were significantly different between groups through univariate and multivariate analyses (P < 0.05).

**Table 1:** Comparisons of patients’ characteristics in training and validation datasets.

Characteristics	Training dataset (n = 120)			Validation dataset (n = 53)		
	MVI (+)	MVI (-)	*P*	MVI (+)	MVI (-)	*P*
Age, years			0.583			0.838
< 35	2	3		1	1	
35-65	28	36		21	22	
≥ 65	27	24		3	5	
Gender			0.283			0.614
Male	49	58		21	22	
Female	8	5		4	6	
BMI			0.652			0.994
< 18.5	5	3		1	1	
18.5-25	30	33		18	20	
≥ 25	22	27		6	7	
α-Fetoprotein			**<0.001**			**<0.001**
< 20 ng/mL	16	38		9	16	
≥20ng/mL	41	25		16	12	
Edmondson-Steiner Grade			0.015			0.478
I	7	11		1	1	
II	30	44		16	22	
III	20	8		8	5	
Cirrhosis of background liver			0.866			0.053
Absent	19	18		5	13	
Present	44	39		19	15	
Serum albumin			0.629			0.028
< 35 g/L	8	7		6	1	
≥35 g/L	49	56		19	27	
Alanine transaminase			0.034			0.224
< 40 U/L	29	44		11	17	
≥ 40 U/L	28	19		14	11	
Aspartate transaminase			**0.018**			**0.043**
< 40 U/L	24	46		11	20	
≥ 40 U/L	33	17		14	8	
Total billrubin			0.522			0.346
< 21 μmol/L	47	49		20	25	
≥ 21 μmol/L	10	14		5	3	
γ-Glutamyltransferase			0.022			0.200
< 60 U/L	6	17		9	15	
≥ 60 U/L	51	46		16	13	
Neutrophils, 10^9/L*	3.50 ± 2.12	3.28 ± 1.25	0.483	3.62 ± 1.18	3.36 ± 0.74	0.142
Lymphocyte, 10^9/L*	1.74 ± 0.60	1.64 ± 0.52	0.337	1.80 ± 0.74	1.52 ± 0.43	<0.001
Hemoglobin, g/L*	141 ± 16	142 ± 13	0.837	147 ± 10	132 ± 10	0.006
Platelet,10^9/L*	182 ± 72	166 ± 66	0.251	208 ± 83	169 ± 24	0.034
NLR			**0.019**			**0.004**
< 1.45	24	40		9	21	
≥ 1.45	33	23		16	7	
Tumor size			0.247			0.001
< 5cm	39	49		5	18	
≥ 5cm	18	14		20	10	
Tumor margin			0.005			0.100
Smooth margin	8	23		2	7	
Non-smooth margin	49	40		23	21	
Enhancement pattern			0.398			0.224
Typical	21	28		14	11	
Atypical	36	35		11	17	
Peritumoral enhancement on artery phase			**0.004**			**0.040**
Absent	27	46		9	18	
Present	30	17		16	10	
Radiologic capsule on delay phase			0.698			0.884
Absent	37	43		12	14	
Present	20	20		13	14	
Peritumoral hypointensity			0.269			0.487
Absent	25	34		11	15	
Present	32	29		14	13	

Unless otherwise noted, data are shown as number of patients, with the percentage in parentheses.

*Data are medians, with interquartile ranges in parentheses.Bold indicate values below 0.05, which are statistically significant.

**Table 2:** Predictive efficacy of the inflammation markers.

Inflammation marker	Threshold	Youden index	Sensitivity	Specificity	*P*
Neutrophil to lymphocyte ratio,NLR	1.45	0.277	0.585	0.692	**0.002**
Systemic Immune Inflammation Index, SII	482.04	0.104	0.280	0.824	0.341
Platelet to lymphocyteratio, PLR	115.89	0.118	0.415	0.703	0.376

Bold indicate values below 0.05, which are statistically significant.

### 3.3 Traditional radiomics feature analysis

For all MRI radiomics features, 982 stable features were retained through the evaluation of consistency (ICCs > 0.75). 4 features in artery phase, 2 features in venous phase, 3 features in delayed phase and 6 features in T2-weighted images were selected by Mann-Whitney U-test, univariate logistic, and LASSO analysis, sequentially (P < 0.05). The lasso dimension reduction analysis of radiomics features was presented in **Supplementary Figure S2**. **Supplementary Figure S3** shows the predictive performance of each feature.

### 3.4 Deep learning feature analysis

A total of 951 deep learning features were preserved following the consistency check (ICCs > 0.75). Following the same method, 2 features in artery phase, 1 feature in venous phase, 1 feature in delayed phase, and 3 features in T2-weighted images were selected. The lasso dimension reduction analysis of deep learning features was shown in **Supplementary Figure S4**. The predictive performance of each characteristic was illustrated in **Supplementary Figure S3**.

### 3.5 Correlation between the NLR and extracted features

**Figure 4:**
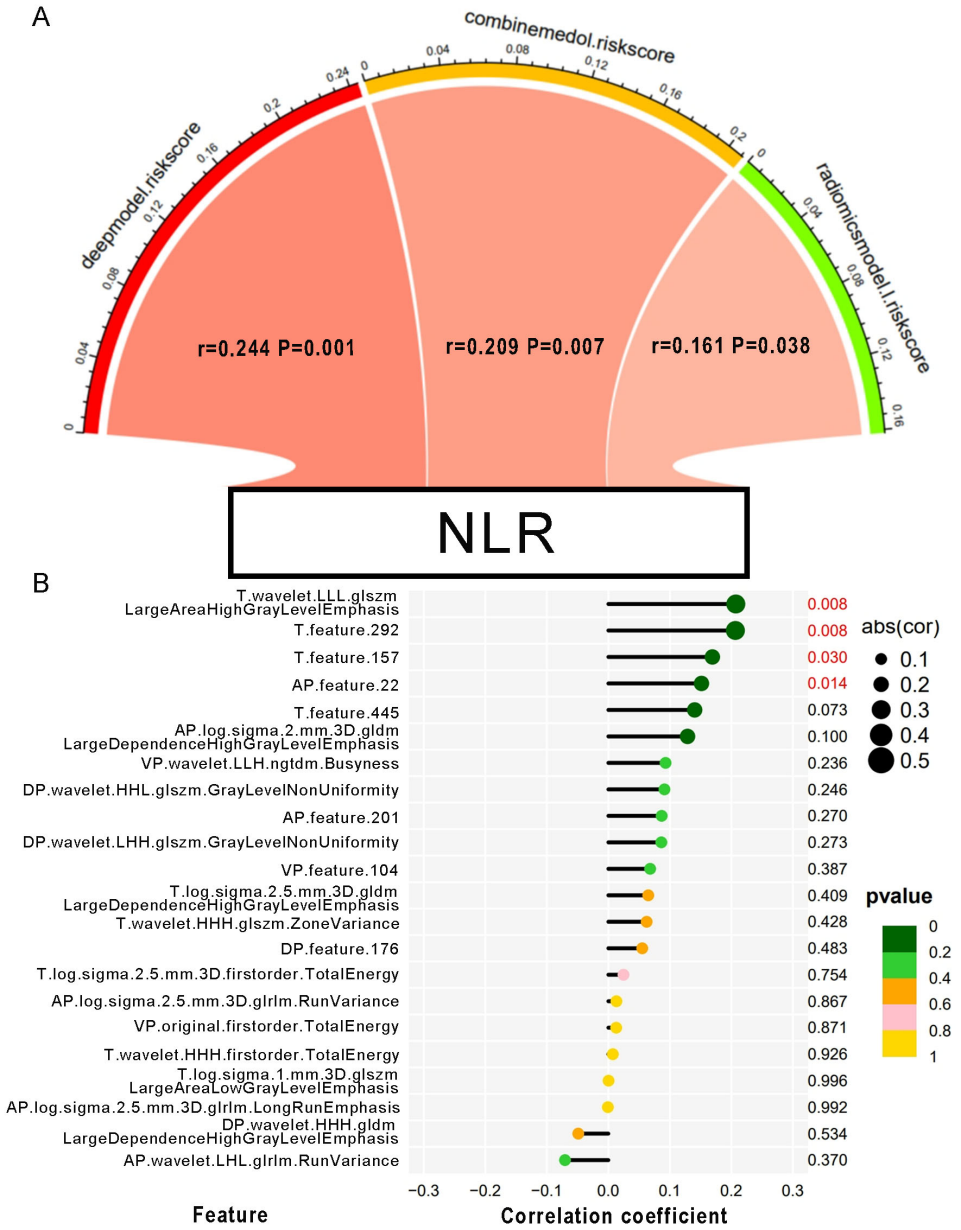
The correlation between the NLR and MRI features. **(A)** Pearson correlation between NLR and the risk scores of each predictive model. The color of the band represented the R-value. **(B)** Correlation of the NLR with every single MVI-related MRI feature. The size of the circle represents the magnitude of the correlation coefficient. The color of the circle indicates the magnitude of the p-value.

The correlation between NLR and radiomics and deep features was tested using Pearson analysis. As shown in **Figure 4**, the result showed that NLR was positively correlated with features like wavelet.LLL.glszm.LargeAreaHighGrayLevelEmphasis radiomics feature, deep feature 292, deep feature 157 in T2 phase and deep feature 22 in artery phase. Furthermore, we discovered the positive relationship between the NLR and 3 predictive model including deep learning model, radiomics model, and combined model (r = 0.244; *P* = 0.001; r = 0.161; *P* = 0.038; r = 0.209; *P* = 0.007). Reasonably, the results indicated that the inflammatory signature could be a good indicator for reflecting inflammation in MRI images and a new biomarker for MVI prediction with radiomics features in HBV-HCC patients.

### 3.6 Model construction and evaluation

#### 3.6.1 Clinical model

**Figure 5:**
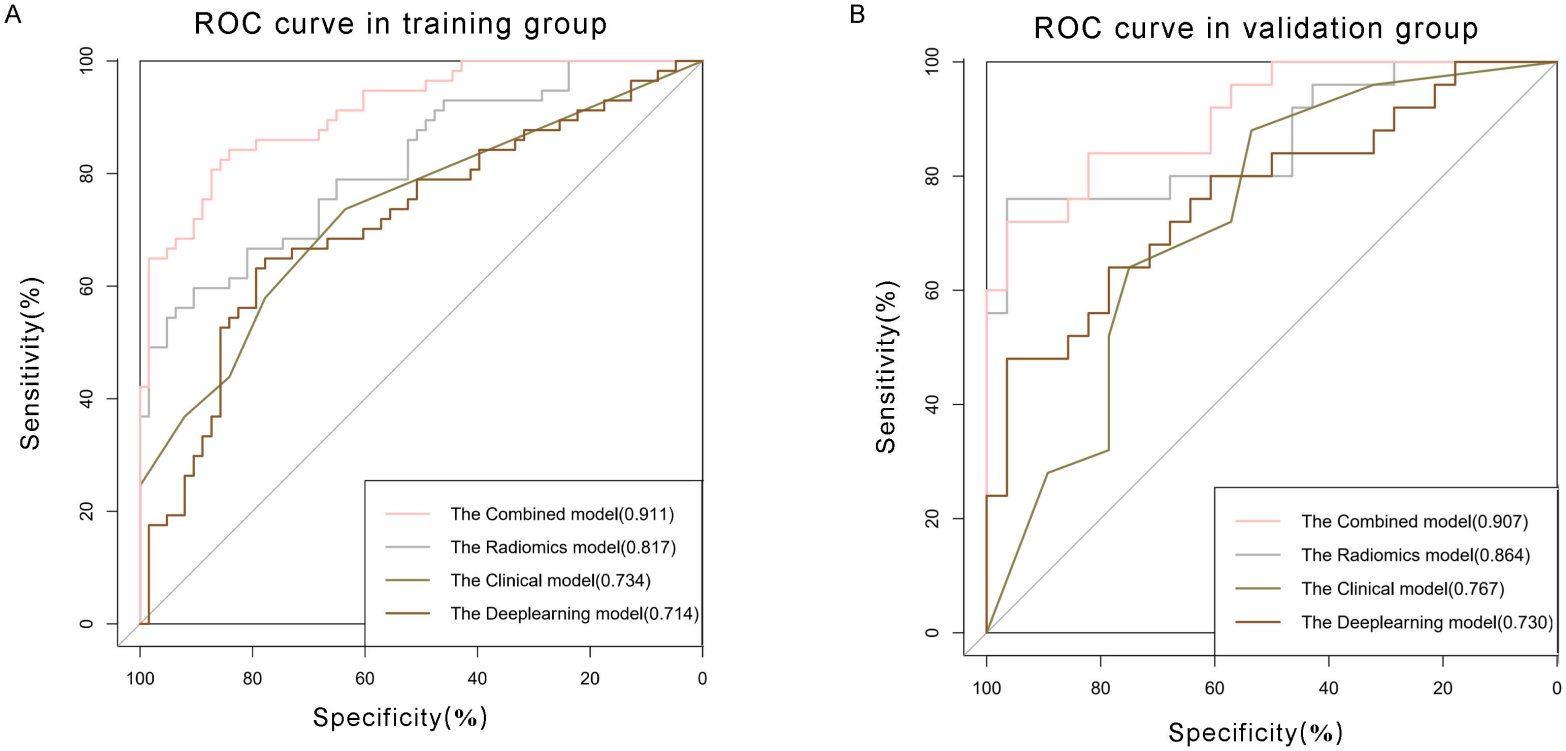
Comparison of receiver operating characteristic (ROC) curves for prediction of microvascular invasion. ROC curves of clinical model (pink curve), radiomics model (grey curve), deep learning model (olive green curve), and combined model (brown curve) in the **(A)** training and **(B)** validation datasets. The X-axis represents the specificity predicted by the model, the Y-axis represents the model’s sensitivity, and the AUC indicates the predictive performance of the predictive model.

The univariate analysis indicated a significant association between MVI and several independent variables, including NLR, serum AFP, AST, and the presence of peritumoral enhancement. Upon multivariate logistic regression analysis, it was ascertained that NLR [odds ratio (OR) 2.837; 95% confidence interval (CI) 1.412-5.703; P = 0.003], serum AFP [OR 3.95; 95% CI 1.936-8.061; P < 0.001], peritumoral enhancement [OR 2.605; 95% CI 1.285-5.283; P = 0.008], and AST [OR 2.916; 95% CI 1.433-5.935; P = 0.003] emerged as independent predictors of MVI, as shown in **Table 3**. These were also effective components in the construction of the clinical model. In conclusion, the AUCs for the clinical model yielded values of 0.734 [95% CI: 0.7024-0.8685] for the training group and 0.767 [95% CI: 0.6778-0.9194] for the validation cohort, respectively (**Figure 5**).

**Table 3:** Logistics regression analysis for MVI.

Characteristic	Hazard ratio	95%CI	*P*
AFP	3.95	1.936-8.061	**<0.001**
NLR	2.837	1.412-5.703	**0.003**
Peritumoral enhancement	2.605	1.285-5.283	**0.008**
AST	2.916	1.433-5.935	**0.003**

Bold indicate values below 0.05, which are statistically significant.

#### 3.6.2 Single radiomics model

Multivariate logistic regression was used to build the model for each MRI phase. The quartet of radiomics models were designated as the arterial, venous+delay, arterial+venous+delay, and T2 model, each predicated on features derived from the arterial phase, portal venous phase, delayed phases, and T2-weighted imaging sequences, respectively. The performance of each model is shown in **Table 4**. Within the training group, the AUCs for the artery, venous+delay, artery+venous+delay, and T2 models were 0.696 [95% CI: 0.6014-0.7910], 0.774 [95% CI: 0.6917-0.8566], 0.779 [95% CI: 0.6978-0.8611], and 0.698 [95% CI: 0.6025-0.7927]. In the validation cohort, the diagnostic efficacy of the radiomics models was quantitatively evaluated. The arterial model demonstrated a notable area under the curve (AUC) of 0.820, with a 95% confidence interval (CI) ranging from 0.7018 to 0.9382. The venous+delay model exhibited an AUC of 0.769 (95% CI: 0.6343-0.9029), reflecting substantial discriminative capability. The arterial+venous+delay model achieved an AUC of 0.803 (95% CI: 0.6844-0.9213), indicating a robust performance in prognostic evaluation. Lastly, the T2 model presented an AUC of 0.724 (95% CI: 0.5731-0.8755), suggesting a respectable level of diagnostic accuracy within the context of the studied parameters. In the training and validation cohorts, the artery model and artery+venous+delay model predicted more accurately than the other radiomic signature models, respectively.

**Table 4:** Predictive efficacy of different radiomics models.

Different models	Training group (n = 120)				Validation group (n = 53)			
	sensitivity	specificity	AUC	AUC (95% CI)	sensitivity	specificity	AUC	AUC (95% CI)
The radiomics model	0.807	0.73	0.858	0.7928-0.9226	0.84	0.786	0.851	0.7401-0.9628
The artery+venous+ delayed model	0.807	0.635	0.779	0.6978-0.8611	0.64	0.857	0.803	0.6844-0.9213
The artery model	0.439	0.905	0.696	0.6014-0.7910	0.857	0.76	0.82	0.7018-0.9382
The venous+delayed model	0.86	0.587	0.774	0.6917-0.8566	0.76	0.75	0.769	0.6343-0.9029
The T2 model	0.386	0.937	0.698	0.6025-0.7927	0.6	0.929	0.724	0.5731-0.8755

**Table 5:** Predictive efficacy of predictive models.

Different models	Training group (n = 120)				Validation group (n = 53)			
	sensitivity	specificity	AUC	(95% CI)	sensitivity	specificity	AUC	(95% CI)
The combined model	0.841	0.842	0.911	0.862-0.961	0.720	0.964	0.907	0.831-0.984
The radiomics model	0.807	0.73	0.858	0.793-0.923	0.84	0.786	0.851	0.740-0.963
The deep learning model	0.649	0.778	0.714	0.620-0.808	0.480	0.964	0.767	0.638-0.897
The clinical model	0.737	0.635	0.734	0.645-0.823	0.88	0.536	0.730	0.593-0.867

#### 3.6.3 Radiomics and deep learning model

The Radiomics model, utilizing features extracted from four distinct MRI phases, yielded an AUC of 0.817, with a 95% CI spanning from 0.742 to 0.892 in the training cohort. And the AUC value of the Radiomics model is 0.864(95% CI: 0.761-0.967) in validation group. Then we applied residual networks (ResNet) to construct a predictive model. This prediction model, which used deep MR image features to forecast MVI, gave back the following performance data. In the training cohort, the model demonstrated a specificity of 0.778 and a sensitivity of 0.649. The AUC for this model was determined to be 0.714, with a 95% CI ranging from 0.620 to 0.808. The AUC for the test cohort was 0.767 (95% CI: 0.637-0.896), while the corresponding specificity and sensitivity were 0.964 and 0.480. The ROC curves of radiomics and deep learning models were presented in **Figure 5**.

#### 3.6.4 Development and validation of the combined model

**Figure 6:**
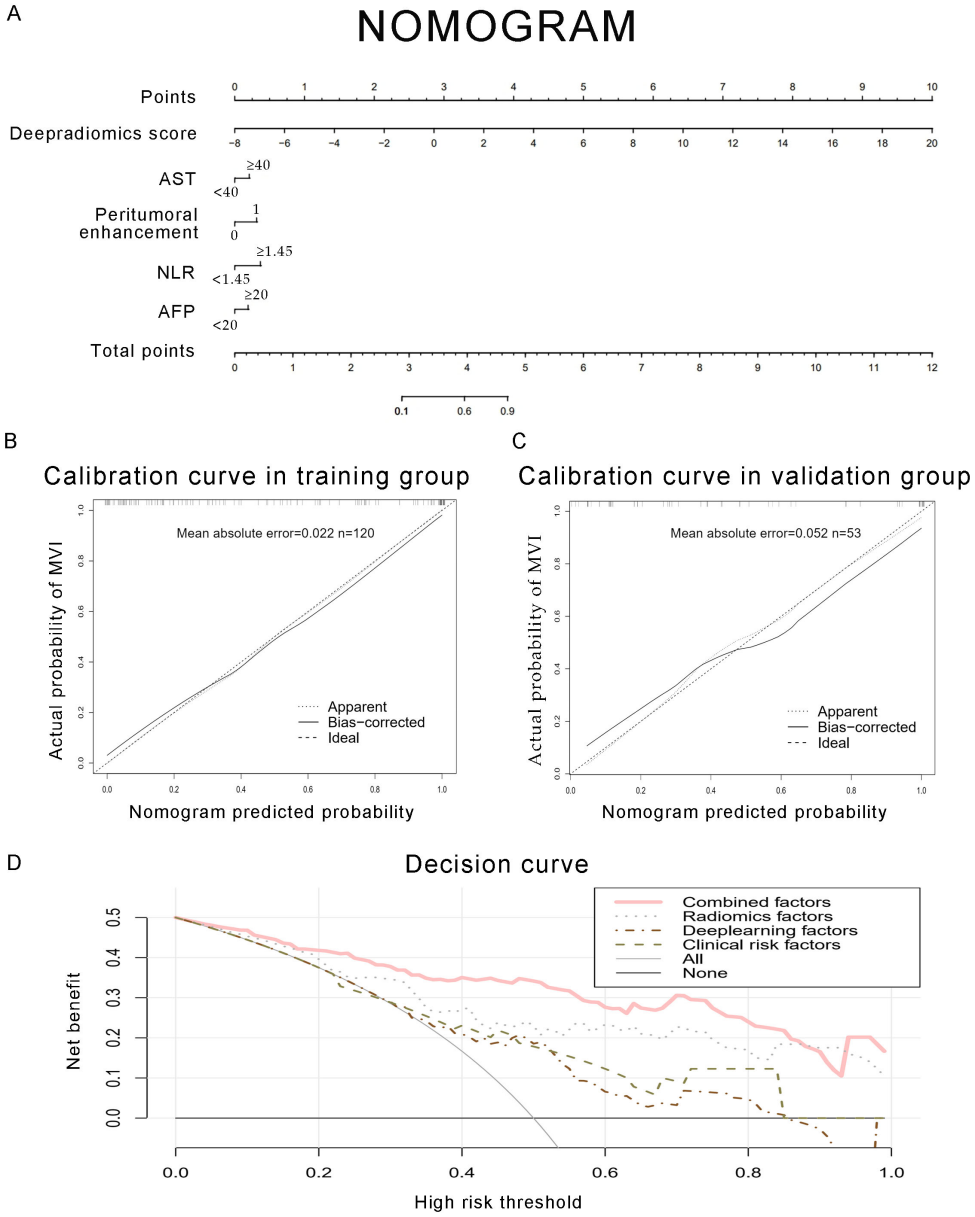
Development of a predictive nomogram for assessing the probabilities of MVI, along with calibration and decision curves. **(A)** A nomogram combining fusion radiomics-deep risk score, inflammation markers (NLR, AST), serum AFP, and radiological factor (peritumoral enhancement). **(B, C)** Calibration curves of the nomogram in the training and validation groups. The X-axis is the nomogram-predicted probability of MVI. The Y-axis is the actual probability of MVI. **(D)** Decision curve of the nomogram for predicting MVI. The pink line represents the expected net benefit per patient derived from the predictive nomogram.

**Figure 7:**
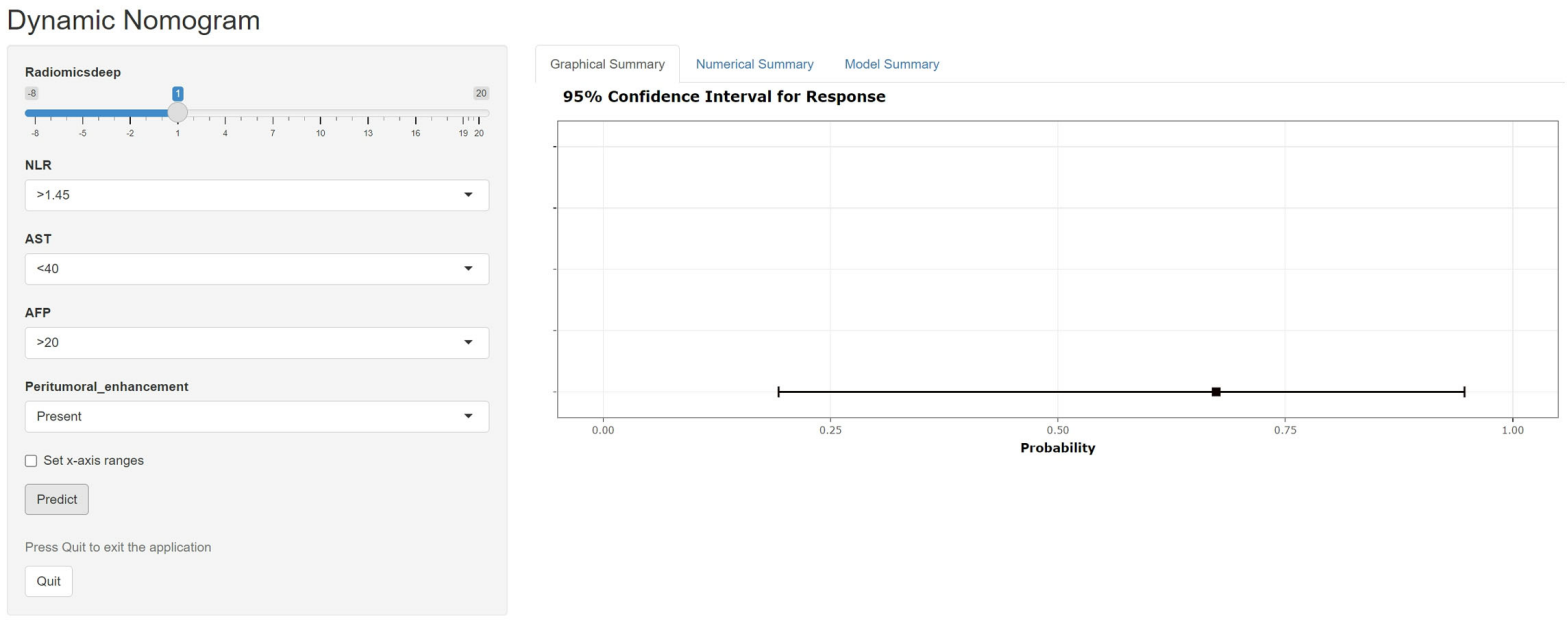
A web-dynamic nomogram for MVI prediction. The MVI probability and 95% confidence interval of an HBV-HCC patient with a radiomics score of 1, NLR over 1.45, AST below 40, serum AFP over 20, and signs of peritumoral enhancement on preoperative MRI were evaluated.

Clinical characteristics, radiomics features and deep learning features were incorporated to construct a Combined model. This MVI predictive model combining all significant independent predictors outperformed other models with an AUC of 0.900(95% CI: 0.8474-0.9532). The combined model was able to predict MVI more accurately. The performance of the model in the training set was characterized by an AUC of 0.911, with a 95% CI ranging from 0.862 to 0.960. It demonstrated a sensitivity of 84.2% and a specificity of 84.1%, as detailed in **Figure 5**. When utilized in the validation group, the model produced an AUC of 0.907, with a 95% confidence interval (CI) from 0.831 to 0.984, with specificity values of 96.4%, sensitivity values of 72.0% respectively. The performance of the combined model, the radiomics model, the deep learning model, and the clinical model is shown in **Table 5**. **Figure 6** depicts the nomogram based on the merged model. The nomogram demonstrated satisfactory predictive accuracy, as reflected by a C-index of 0.926, with a 95% CI ranging between 0.881 and 0.969, in the training group. Similarly, in the validation group, the C-index was an impressive 0.917, with a 95% CI of 0.846 to 0.988. The calibration curves (**Figure 6**) illustrated that the nomogram’s predicted probabilities exhibited a high degree of correlation with the actual occurrences of MVI in both the training and validation groups. In the training group, the calibration was statistically significant with a P-value of 0.022, while in the validation group, the result nearly reached statistical significance with a P-value of 0.052. Decision curve for the nomogram was demonstrated in **Figure 6**. Considering the findings, a dynamic online tool (https://zhongyun.shinyapps.io/HBV-HCC_MVI_NOMOGRAM/) has been created for forecasting the likelihood of microvascular invasion in individuals diagnosed with HBV-related HCC (**Figure 7**).

## 4 Discussion

The role of systemic inflammation in the pathogenesis of hepatocellular carcinoma (HCC) in the context of hepatitis B virus (HBV) infection has been extensively examined (18). The design of this retrospective study aimed to construct and confirm a novel nomogram that incorporates preoperative indicators, including markers of blood-cell-mediated inflammation and radiomics-derived inflammation, for the prognostication of MVI in individuals with HBV-HCC. In contrast to earlier findings, we introduced MRI deep radiomics features to construct a new predictive model called the deep radiomics model.

In the systemic milieu induced by the tumor, a myriad of inflammatory cytokines contribute to the invasion of HCC cells and the progression of metastases (20). Inflammation markers derived from blood cells, such as the neutrophil-to-lymphocyte ratio (NLR), systemic immune-inflammation index (SII), and aspartate aminotransferase-to-platelet ratio index (APRI), represent ratios that correlate various cellular proportions implicated in the inflammatory response. In our investigation, patients with HBV-HCC exhibited elevated levels of these inflammatory indices. Hernandez-Ainsa et al. have reported an association between elevated values of NLR, PLR, and SII with increased tumor invasiveness, lower differentiation grades, and diminished overall survival rates (24). In the cascade of cancer cell metastasis, neoplastic cells first infiltrate the adjacent microvasculature, encompassing the intratumoral vascular compartments, before propagating through these microvascular structures. A strong relationship between MVI and tumor recurrence and survival after transplant and surgical resection has been reported in recent retrospective analysis (25, 26). Consequently, this gives rise to the hypothesis that the presence of extratumoral MVI constitutes a significant risk factor for the recurrence of the tumor (27).

The NLR is an inflammatory marker that has been studied as a predictive indication of recurrence and survival in HCC patients (28). Higher NLR correlates with a heightened infiltration of inflammatory cells and an enhanced secretion of inflammatory cytokines, leading to a proliferation of neutrophil populations (29). According to the findings of our study, NLR was significantly associated with MVI, showing an increasing value at a higher incidence of MVI. This phenomenon could be attributed to the substantial release of neutrophils, which amplifies the potential for tumor progression and vascular invasion through the upregulation of vascular endothelial growth factor and additional pro-inflammatory cytokines (30).

The AST is an important enzyme in the liver, which is mainly present in the mitochondria of liver cells. In highly proliferative cancer tissues, the level of AST is more frequently activated. Ellen Friday has found that the increase in AST might be related to the tumor growth and progression. Our research has also demonstrated that the higher level of AST was associated with MVI in HCC patients.

As a conventional clinical biomarker in HCC, our observations corresponded with an established trend, wherein serum AFP paralleled the likelihood of MVI. These inflammatory indices would probably identify high-risk populations and improve our screening methods. Using these biomarkers, we developed a clinical model that combined NLR, AFP, and AST levels to identify HCC with MVI, achieving an accuracy with an AUC of 0.767. Our results are in line with those reported by Hidetoshi Nitta, indicating that the nomogram offers a reliable method for predicting extra tumoral MVI in patients undergoing hepatic resection or liver transplantation (27).

Prior studies have noted the importance of preoperative tumor images in tumor immune biology and immunotherapy response. Traditional radiomics leverages advanced computing tools to extract deeper and more granular data from imaging (31). As Marius E. Mayerhoefer mentioned, radiomics models based on large high-quality and well-curated data sets have a better performance, so we tried to use radiomics features to construct a predictive model (32). In the context of this retrospective analysis, we identified 15 radiomic features correlated with MVI in HCC from T2-weighted phase images and standard triphasic phase images. These features were subsequently utilized for the construction of a radiomics score. In these 15 texture parameters, GLRLM_Long RunEmphasis, NGTDM_Busyness, GLDM_LargeDependenceHighGrayLevelEmphasis and Firstorder_TotalEnergy were the most significant difference between the two groups in 4 phases, respectively. LargeDependenceHighGrayLevelEmphasis measures the joint distribution of large dependence with higher gray-level values, with a higher value indicating more homogeneity (33). This parameter in venous phase has strongest predictive performance. The increased heterogeneity observed in the MVI-positive group within MRI scans can be ascribed to the presence of a greater diversity of atypical vessels, a higher incidence of necrotic vessels stemming from rapid tumor growth, and a more heterogeneous internal structure of the tumors (34). The inflammatory reaction within tumor microenvironment causes the proliferation of abnormal blood vessels and the necrosis of the tumor tissue, resulting in the appearance of uneven tumor internal structure in images.

With the development of high-throughput computing technology and artificial intelligence, deep image features are obtained by neural convolutional network algorithm such as CNN. In contrast to conventional radiomics methods, the image features are directly extracted from the deep neural network, hence ensuring that the deep learning radiomics extraction technique remains error-free (35). After extracting MRI deep features with 3D ResNet and LASSO analysis, 7 deep features were selected as MVI risk factors to construct a deep radiomics prediction model. We developed models based on radiomics features and deep learning features for predicting MVI in training dataset, with AUCs of 0.817 and 0.714, respectively. And validated in validation dataset, the AUCs of the two models were 0.864 and 0.730 respectively. Two models performed well in terms of MVI prediction.

The literature has documented that radiomics features might serve as indicators of the tumor microenvironment in patients (36). The occurrence of MVI might be related to the local inflammation in the tumor microenvironment (37). The radiomics model has a good prediction effect on MVI, and we speculate that there is a correlation between radiomics and inflammatory indicators. Finally, we also proved that radiomics features were positively correlated with NLR, which reflects inflammation. Simultaneously, it was confirmed that the risk scores of the deep and radiomics models were positively connected with NLR. As both preoperative examinations, we infer that the clinical and MRI indices could reflect the pro/antitumorigenic inflammatory status in two different ways. Hence, it could conceivably be hypothesized that clinical characteristics plus MRI signatures can improve the predictive value for MVI. We constructed a combined model, the result is just as what we have supposed. Our study supported the findings of Wenjun Yao, which indicated that a combined model leveraging both clinical and radiomic signatures delivered superior predictive performance, as evidenced by a high AUC, and more effectively differentiated MVI when compared to models based on clinical or radiomic markers alone (38).

Our combined model achieved an AUC of 0.907, sensitivity of 72.0%, and specificity of 96.4%. These metrics were comparable to those reported by Yang et al., who reported an AUC of 0.861, using a similar MRI-based approach (13). Notably, the AUCs of the clinical model and the radiomics model were similar to those obtained in their study, but the combined model yielded a higher AUC. This underscores the effectiveness of deep learning features in accurately identifying MVI. When compared to the study by Zhou et al., which employed 3D convolutional neural networks on contrast-enhanced MRI to predictive MVI in HCC, our study also utilized advanced feature extraction method (39). This led to an AUC improvement, highlighting the superiority of our approach in capturing significant features linked to MVI. Additionally, the interpretability of our model was enhanced through the analysis of the correlation between radiomic features and NLR, providing insights into the contribution of individual radiomic features. This transparency is a significant advancement over earlier studies, such as that by Mu He, where the model incorporating neutrophils lacks interpretability (40).

Our analysis extended to examining the performance of radiomics models in different imaging phases, revealing that the model integrating venous and delayed phase images yielded better predictive accuracy than the model based on arterial phase imaging. Hypervascularity during the arterial phase of enhancement and wash-out during the portal phase correspond to the Barcelona criteria for HCC (41). The high AUC of the model may be due to the enhancement patterns and “wash-out” characteristics typical of HCC seen in imaging studies (42). Even though deep learning model’s prediction performance was lower than radiomics model, there was still a high positive correlation between some deep learning features and NLR. In the radiomics analysis of HBV-related HCC, which was closely related to inflammation, we speculated deep learning features were a good complement to traditional radiomics analysis. The AUCs of the combined model also corresponded to our thoughts, and the C-index and the decision curve both verified the good predictive performance of the combined model.

This study is subject to certain limitations, including its single-center design and retrospective nature. Furthermore, the sample size is relatively small, which may affect the generalizability of the findings. To develop and validate an accurate prediction model for microvascular invasion (MVI) grading, additional research involving larger populations is imperative.

## 5 Conclusion

In conclusion, the combined model achieves satisfactory preoperative prediction of MVI in HBV-related HCC. In other models, the radiomics model has good prediction performance, and deep learning features are a better complement for MVI prediction. NLR is positively correlated with MRI features. The nomogram based on clinical risk factors and MRI characteristics would help clinicians and patients make an individualized risk assessment of MVI.

## Data Availability

The raw data supporting the conclusions of this article will be made available by the authors, without undue reservation.
