# Supplementary material for: Assessing microvascular invasion in HBV-related hepatocellular carcinoma: an online interactive nomogram integrating inflammatory markers, radiomics, and convolutional neural networks
Source: Front Oncol. 2024 Sep 16;14:1401095. doi: 10.3389/fonc.2024.1401095 (PMC11439624; doi:10.3389/fonc.2024.1401095)
Supplement: Supplementary file 1 [file DataSheet1.pdf]

TableS1. Comparisons of patients characteristics in training and validation datasets

Characteristics	Training dataset (n = 120)	Validation dataset (n = 53)	*P*
MVI			0.968
Positive	57	25	
Negetive	63	28	
Age, years			**0.002**
< 35	5	2	
35-65	64	43	
⩾ 65	51	8	
Gender			0.151
Male	13	43	
Female	13	10	
BMI			0.061
< 18.5	8	2	
18.5-25	63	38	
⩾ 25	49	13	
α-Fetoprotein			0.792
< 20 ng/mL	54	25	
⩾20ng/mL	66	28	
Edmondson-Steiner Grade			0.100
I	18	2	
II	74	38	
III	28	13	
Cirrhosis of background liver			0.625
Absent	37	18	
Present	83	34	
Serum albumin			0.898
< 35 g/L	15	7	
⩾35 g/L	105	46	
Alanine transaminase			0.325
< 40 U/L	73	28	
⩾ 40 U/L	47	25	
Aspartate transaminase			**0.014**
< 40 U/L	70	31	
⩾ 40 U/L	50	22	
Total billrubin			0.444
< 21 μmol/L	96	45	
⩾ 21 μmol/L	24	8	
γ-Glutamyltransferase			**<0.001**
< 60 U/L	23	24	
⩾ 60 U/L	97	29	
Neutrophils, 10^9/L*	3.39±1.76	3.18±1.37	0.422
Lymphocyte, 10^9/L*	1.68±0.56	1.71±0.70	0.816
Hemoglobin, g/L*	141±14	140±15	0.370
Platelet,10^9/L*	175±70	183±73	0.461
Tumor size			**<0.001**
< 5cm	88	23	
⩾ 5cm	32	30	
Tumor margin			0.203
Smooth margin	31	9	
Non-smooth margin	89	44	

Enhancement pattern			0.437
Typical	49	25	
Atypical	71	28	
Peritumoral enhancement on artery phase			0.225
Absent	73	27	
Present	47	26	
Radiologic capsule on delay phase			**0.028**
Absent	80	26	
Present	40	27	
Peritumoral hypointensity			0.989
Absent	59	26	
Present	61	27	
