# Supplementary figures and images for: Assessing microvascular invasion in HBV-related hepatocellular carcinoma: an online interactive nomogram integrating inflammatory markers, radiomics, and convolutional neural networks
Source: Front Oncol. 2024 Sep 16;14:1401095. doi: 10.3389/fonc.2024.1401095 (PMC11439624; doi:10.3389/fonc.2024.1401095)

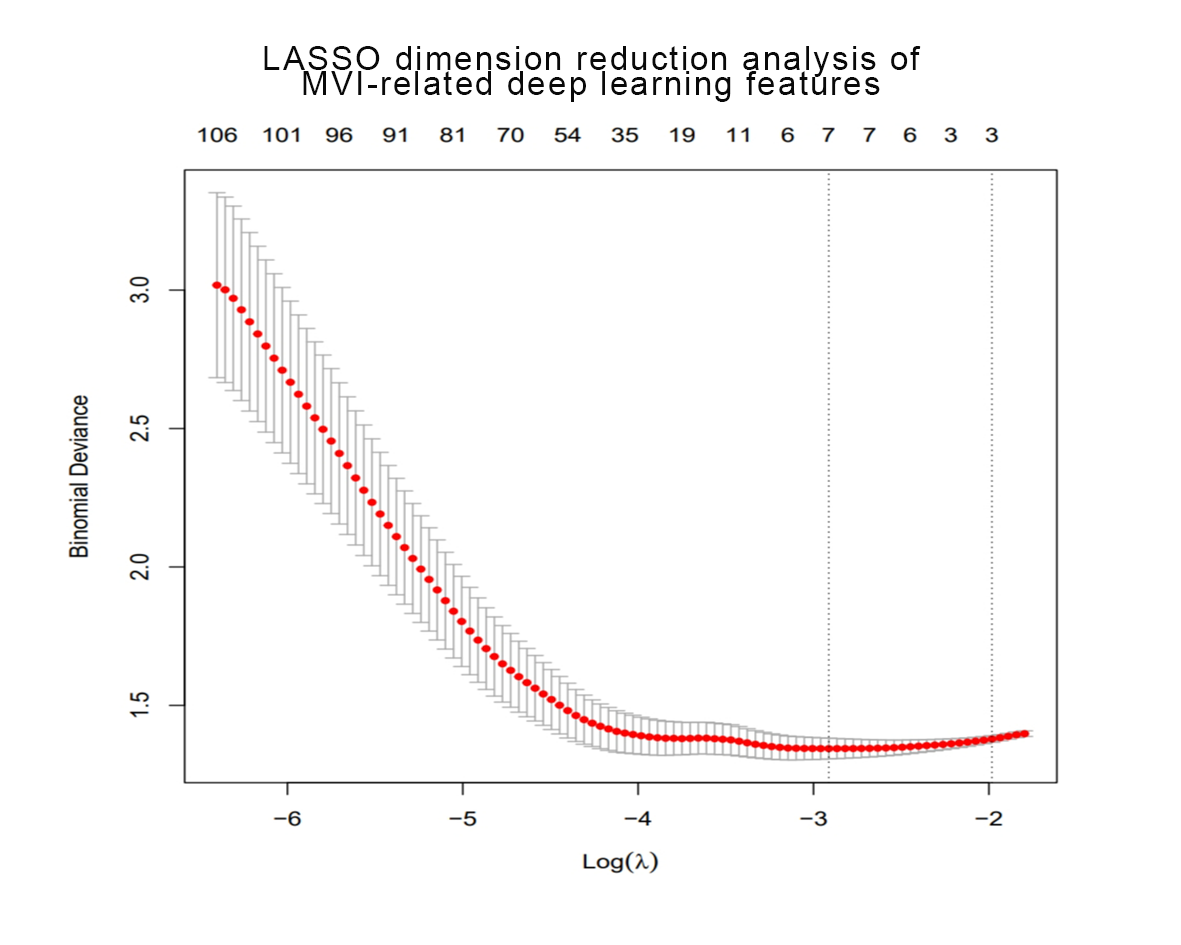

Supplement: Supplementary file 2 [file Image1.tif]

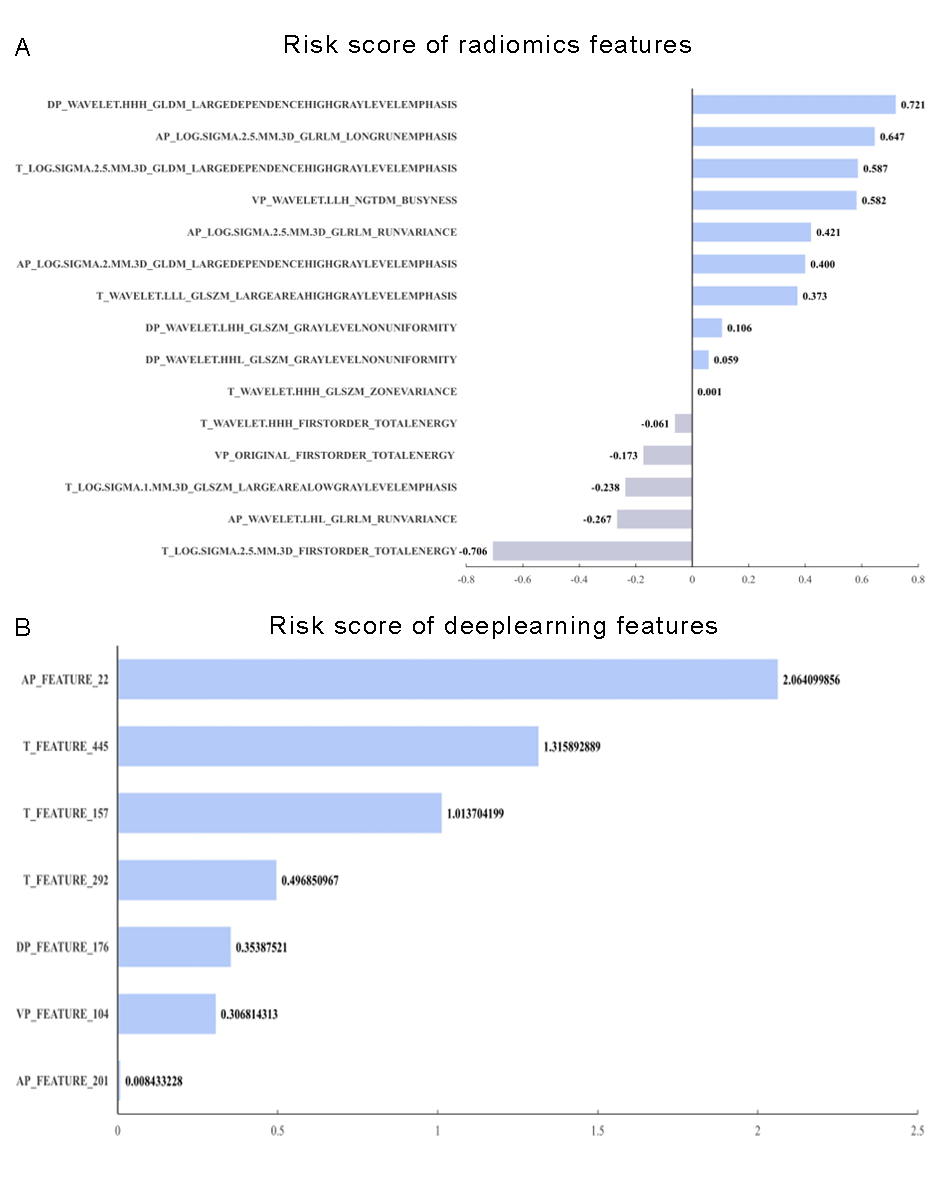

Supplement: Supplementary file 3 [file Image2.tif]

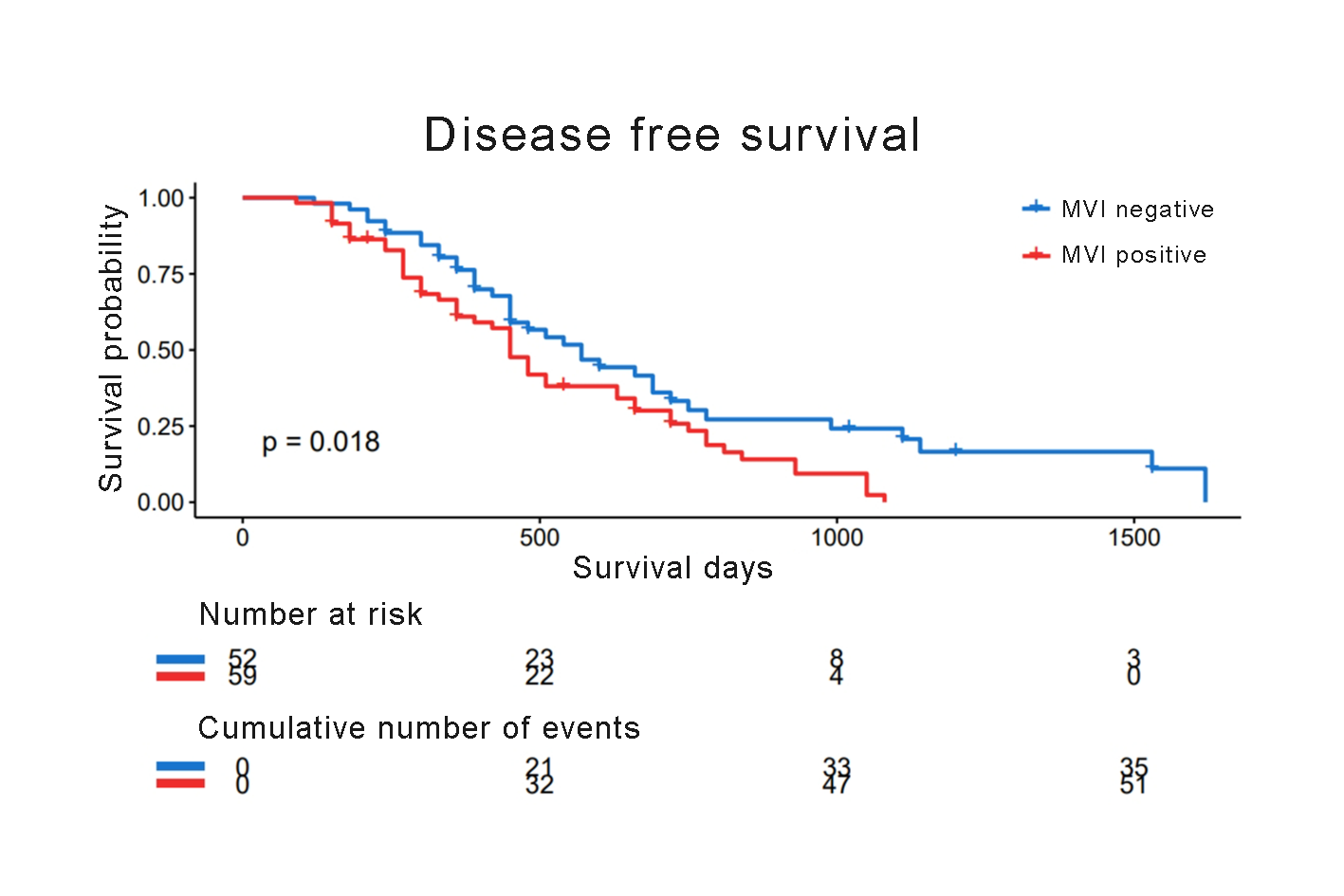

Supplement: Supplementary file 4 [file Image3.tif]

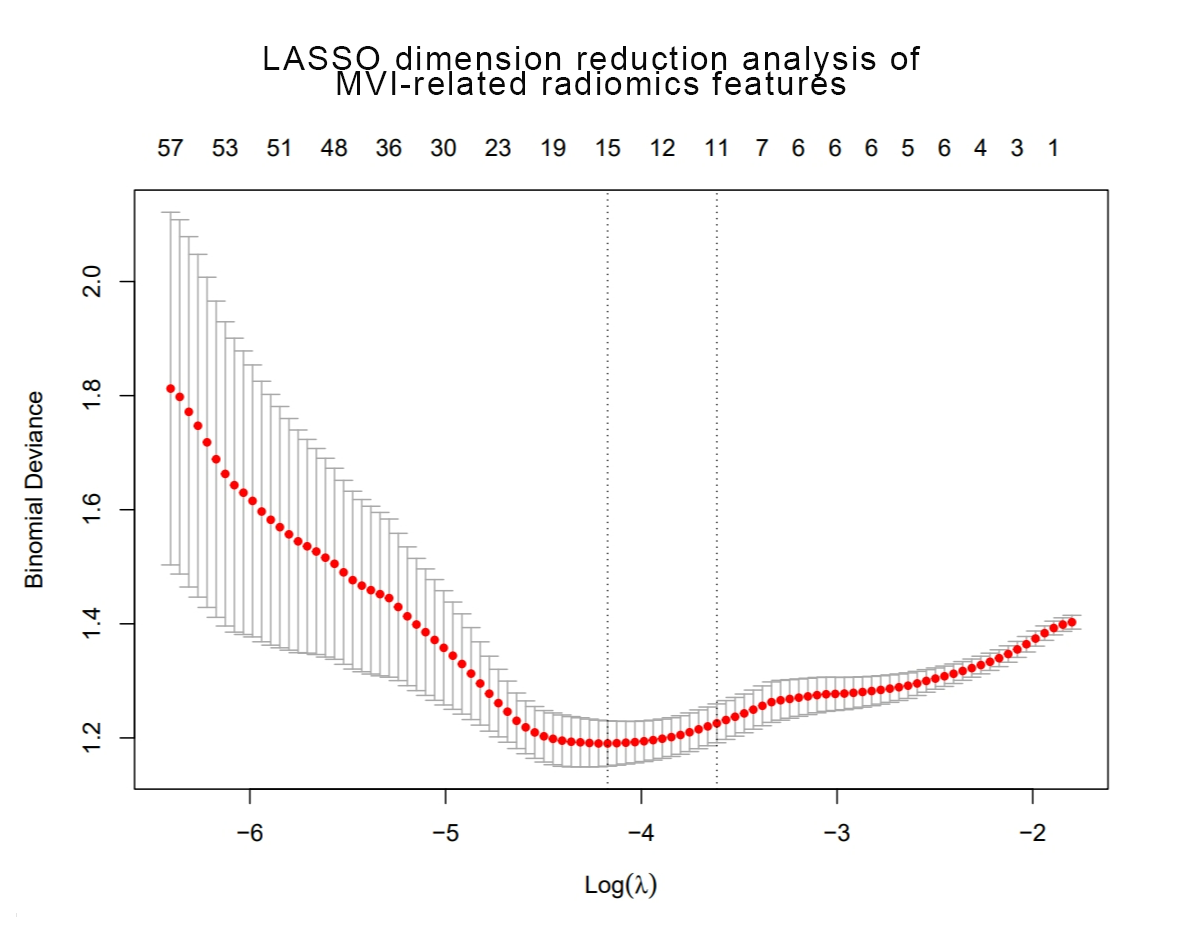

Supplement: Supplementary file 5 [file Image4.tif]
